# *In vitro *microbiological evaluation of 1,1'-(5,5'-(1,4-phenylene)*bis*(3-aryl-1*H*-pyrazole-5,1-(4*H*,5*H*)-diyl))diethanones, novel *bis*acetylated pyrazoles

**DOI:** 10.1186/2191-2858-1-8

**Published:** 2011-09-20

**Authors:** Vijayakumar Kanagarajan, Muthuvel Ramanathan Ezhilarasi, Mannathusamy Gopalakrishnan

**Affiliations:** 1 Energetics Research Institute, Nanyang Technological University, 50, Nanyang Avenue, Singapore-639 798, Republic of Singapore; 2 Synthetic Organic Chemistry Laboratory, Department of Chemistry, Annamalai University, Annamalainagar 608 002, Tamil Nadu, India

**Keywords:** *bis*acetylated pyrazoles, *in situ *acetylation, antibacterial activity, antifungal activity; antitubercular activity

## Abstract

Novel 1,1'-(5,5'-(1,4-phenylene)*bis*(3-aryl-1*H*-pyrazole-5,1-(4*H*,5*H*)-diyl))diethanones **7-12 **were tested for their antimicrobial activity by disc diffusion and twofold serial dilution method against the tested bacterial and fungal strains. Compounds **7 **against *Micrococcus luteus*, **8 **against *β-Heamolytic streptococcus, M. luteus, Klebsiella pneumonia, Microsporum gypseum*, **9 **against *Staphylococcus aureus, Shigella flexneri, Vibreo cholerae, Pseudomonas aeruginosa, Aspergillus flavus, Mucor indicus*, **10 **against *Salmonella typhii, S. flexneri, M. gypseum*, **11 **against *K. pneumonia, M. gypseum*, **12 **against *K. pneumonia*, and *M. gypseum *show superior zone of inhibitions and exhibited excellent antibacterial and antifungal activities at a MIC value of 6.25 μg/mL. Moreover, all the tested compounds **7-12 **revealed promising antitubercular activity against *Mycobacterium tuberculosis *H_37_Rv and INH-resistant *M. tuberculosis*. Compounds **8 **against *M. tuberculosis *and **11 **against INH-resistant *M. tuberculosis *exhibited the percentage of reduction in RLU at 89 and 85%, respectively.

## 1. Introduction

*Mycobacterium tuberculosis *(MTB) is a pathogenic bacterial species in the genus *Mycobacterium *and is the causative agent of most cases of tuberculosis. Tuberculosis is a common and often deadly infectious disease in humans [1,2]. Tuberculosis is the most common opportunistic disease in persons infected with human immunodeficiency virus [3]. The genome of MTB is rich in lipid-metabolizing and P450 enzymes. The cell envelope of MTB is unique and is associated with its pathogenicity [4]. Mycolic acids are the major constituents of the protective barrier of cell envelope of MTB and are essential for survival, virulence, and antibiotic resistance [5]. Inhibitors of mycolic acid biosynthesis, such as isoniazid (INH), ethambutol (EMB), and pyrazinamide (PZA), are still in the frontline of antitubercular drugs [6].

**Scheme 1:**
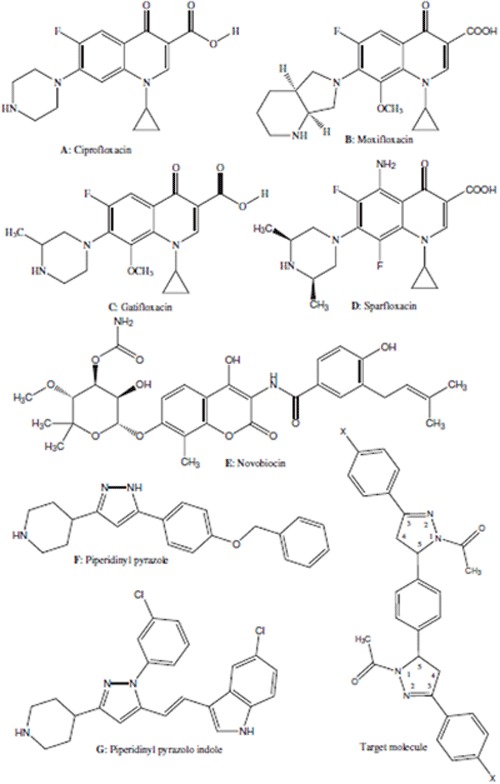
**Structure of novel antitubercular agents**.

The discovery of the norfloxacin plays an important role in structure-activity relationships analysis of the fluoroquinolonic nucleus **A-D**, (Scheme 1) which led to the development of new derivatives with better solubility, higher antimicrobial activity, prolonged serum half-life, fewer adverse side effects, and both oral and parenteral routes of administration [7-9]. Naturally occurring bacterial DNA gyrase inhibitor such as novobiocin, a coumarin derivative **E **(Scheme 1), is known as antibacterial agents [10]. The coumarin drug inhibits ATPase activity of DNA gyrase by competing with ATP for binding to the subunit B of the enzyme. Owing to side effects, no pharmaceutically useful drug has been derived from the coumarins [11]. Although huge efforts have been dedicated to find a potent antibacterial agents that can overcome bacterial resistance, promising lead structures of DNA gyrase and topoisomerase IV enzyme inhibitors with novel mechanisms of action have not been found [12]. This reflects the inherent difficulties associated with the discovery and clinical testing of new candidates and the lack of significant pharmaceutical industry research in this area. Hence, the discovery and development of new drugs that effectively combat TB are accorded a great importance. In recent years, interest in pyrazoles has increased significantly because of their proven usefulness as intermediates in the preparation of new pharmaceuticals and agrochemicals [13-15]. Also, pyrazole derivatives **F **and **G **(Scheme 1) were identified as a new class of DNA gyrase and topoisomerase IV enzyme inhibitors [16]. Besides these, amides are well known for their therapeutic values since the amide group is an important pharmacophore. Antibiotics such as penicillins and cephalosporins have an amide group. The resistance toward available drugs is rapidly becoming a major worldwide problem. The necessity to design new compounds to overcome this resistance has become one of the most important areas of research today. Owing to our interest in synthesizing fascinating biologically active structurally diverse heterocycles [17-20], we recently reported the clean production of 1,1'-(5,5'-(1,4-phenylene)*bis*(3-aryl-1*H*-pyrazole-5,1-(4*H*,5*H*)-diyl))diethanones, a novel series of *bis *pyrazole derivatives using sodium acetate/acetic anhydride triggered by ultrasound irradiation [21], which accelerated the chemical reaction and mass transferred via the process of acoustic cavitation [22]. Extending the research in this area, we decided to investigate the antibacterial, antifungal, and antitubercular activities of the target compounds with the hope to develop some promising antimicrobial and antimycobacterial agents.

## 2. Experimental

### 2.1 Chemistry

Performing TLC assessed the reactions and the purity of the products. All the reported melting points are taken in open capillaries and were uncorrected. Sonication is performed on a Life Care-Fast Ultrasonic system (Life Care Equipments Pvt. Ltd., Mumbai, India) operating at a frequency of 45 kHz. The reaction flask is located in the maximum energy area in the bath and the addition or removal of water controlled the temperature of the water bath. IR spectra are recorded in KBr (pellet forms) on a Thermo Nicolet-Avatar-330 FT-IR spectrophotometer (Thermo Fisher Scientific Inc., Waltham, MA, US) and note worthy absorption values (cm^-1^) alone are listed. ^1^H and ^13^C NMR spectra are recorded at 400 and 100 MHz, respectively, on Bruker AMX 400 NMR spectrometer (Bruker Biospin International, Ag, Aegeristrasse, Switzerland) using C*D*Cl_3 _as solvent. The ESI +ve MS spectra are recorded on a Varian Saturn 2200 MS spectrometer (Varian Inc., Palo Alto, USA). Satisfactory microanalyses are obtained on Carlo Erba 1106 CHN analyzer (Thermo Fisher Scientific Inc., Waltham, MA, US). By adopting the literature precedent, *bis *chalcones **1-6 **[23] and 1,1'-(5,5'-(1,4-phenylene)bis(3-aryl-1H-pyrazole-5,1-(4H, 5H)-diyl))diethanones **7-12 **[21] are prepared.

### 2.2. Microbiology

All the clinically isolated bacterial strains namely *Staphylococcus aureus, β-Heamolytic streptococcus, Micrococcus luteus, Bacillus subtilis, Salmonella typhii, Shigella flexneri, Vibreo cholerae, Escherichia coli, Pseudomonas aeruginosa, Klebsiella pneumonia*, MTB H_37_Rv, INH-resistant MTB and fungal strains namely *Aspergillus flavus, Aspergillus niger, Mucor indicus, Rhizopus arrhizus*, and *Microsporum gypsuem *are obtained from the Faculty of Medicine, Annamalai University, Annamalainagar 608 002, Tamil Nadu, India.

### 2.3. *In vitro *antibacterial and antifungal activity by disc diffusion method

The *in vitro *activities of the compounds were tested in Sabourauds dextrose broth (SDB) (Hi-media, Mumbai) for fungi and nutrient broth (NB) (Hi-media, Mumbai) for bacteria by the disc diffusion method following the reported method [24]. The respective hydrochlorides of the test compounds **7-12 **were dissolved in water to obtain 1 mg/mL stock solution and the different concentrations (100, 200, 500 ppm) were prepared from the stock solution. Seeded broth (broth-containing microbial spores) was prepared in NB from 24-h-old bacterial cultures on nutrient agar (Hi-media, Mumbai) at 37 ± 1°C while fungal spores from 1 to 7-day-old Sabourauds agar (Hi-media, Mumbai) slant cultures were suspended in SDB. Sterile paper disc of 5-mm diameter was saturated with the three different concentrations and such discs were placed in each seeded agar plates. The petri plates were incubated in BOD incubator (Sigma Instruments, Chennai, India) at 37°C for bacteria and at 28°C for fungi. The zone of inhibition was recorded by visual observations after 24 h of inhibition for bacteria and after 72-96 h of inhibition for fungi. Moreover, the zone of inhibition was measured by excluding the diameter of the paper disc. Ciprofloxacin was used as standard for bacteria and fluconazole as standard for fungi under analogous conditions.

### 2.4. *In vitro *antibacterial and antifungal activity by twofold serial dilution method

MIC in μg/mL values was carried out by twofold serial dilution method [25]. The respective test compounds **7-12 **were dissolved in dimethyl sulphoxide (DMSO) to obtain 1 mg/mL stock solution. Seeded broth (broth-containing microbial spores) was prepared in NB from 24-h-old bacterial cultures on nutrient agar (Hi-media, Mumbai) at 37 ± 1°C while fungal spores from 1 to 7-day-old Sabourauds agar (Hi-media, Mumbai) slant cultures were suspended in SDB. The colony forming units (cfu) of the seeded broth were determined by plating technique and adjusted in the range of 10^4^-10^5 ^cfu/mL. The final inoculums size was 10^5 ^cfu/mL for antibacterial assay and 1.1-1.5 × 10^2 ^cfu/mL for antifungal assay. Testing was performed at pH 7.4 ± 0.2 for bacteria (NB) and at a pH 5.6 for fungi (SDB). Exactly 0.4 mL of the solution of test compound was added to 1.6 mL of seeded broth to form the first dilution. One milliliter of this was diluted with a further 1 mL of seeded broth to give the second dilution and so on till six such dilutions were obtained. A set of assay tubes containing only seeded broth was kept as control. The tubes were incubated in BOD incubators at 37 ± 1°C for bacteria and 28 ± 1°C for fungi. MICs were recorded by visual observations after 24 h (for bacteria) and 72-96 h (for fungi) of incubation. Ciprofloxacin was used as standard for bacteria studies and fluconazole was used as standard for fungal studies.

### 2.5. *In vitro *antitubercular activity by luciferase reporter phage assay method

The preliminary antitubercular activity screening was conducted against *M. tuberculosis *H_37_Rv, INH-resistant *M. tuberculosis *by luciferase reporter phage assay method [26] at two different concentrations (1.00 and 2.00 mg/mL). Fifty microliter bacterial suspension equivalent to MacFarlands No. 2 standard was added to 400 mL of G7H9 with and without the test compound. For each sample, two drug-free controls and two drug concentrations were prepared and this setup was incubated for 72 h at 37°C. After incubation, 50 mL of the high titer Luciferase reporter phage (PhAE129) and 40 mL of 0.1 M CaCl_2 _were added to all the vials and this setup was incubated at 37°C for 4 h. After incubation, 100 mL of the mixture was taken from each tube into a luminometer cuvette and equal amount of working D-Luciferin (0.3 mM in 0.05 M sodium citrate buffer, pH 4.5) solution was added. The RLU was measured after 10 s of integration in the Luminometer (Monolight 2010, Pegasus Scientific Inc., Rockvillae, USA). Duplicate readings were recorded for each sample and the mean was calculated. The percentage reduction in the RLU was calculated for each test sample and compared with control. The experiment was repeated when the mean RLU of the control was less than 1,000.

## 3. Results and discussion

### 3.1. Chemistry

**Scheme 2:**
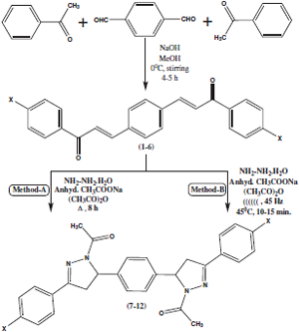
**Synthesis of 1,1'-(5,5'-(1,4-phenylene)bis(3-aryl-1H-pyrazole-5,1-(4H,5H)-diyl))diethanones under thermal and sonication methods using anhydrous sodium acetate/acetic anhydride**.

Synthesis of 1,1'-(5,5'-(1,4-phenylene)*bis*(3-aryl-1*H*-pyrazole-5,1-(4*H*,5*H*)-diyl))diethanones **7-12 **is carried out in excellent yields (Scheme 2 and Table 1) by the reaction of *bis *chalcones **1-6 **with hydrazine hydrate catalyzed by anhydrous sodium acetate/acetic anhydride under ultrasonic irradiation method at 45°C within 10-20 min. It has been observed in the traditional classical method, the reaction mixture of *bis *chalcones **1-6 **with hydrazine hydrate catalyzed by anhydrous sodium acetate in refluxing acetic anhydride for 5-8 h yield compounds **7-12 **in moderate yields. However, when this reaction is performed under sonication method [27], the reaction takes place rapidly within 10-20 min with excellent yields (Table 1). In this study, acetic anhydride is the best solvent for the facile synthesis of *bis *pyrazoles, **7-12 **in excellent yields without any solubility problem. In addition, *in situ *acetylation occurs in the course of the reaction because of solvent, acetic anhydride under the reaction conditions. The structures of the synthesized 1,1'-(5,5'-(1,4-phenylene)*bis*(3-aryl-1*H*-pyrazole-5,1-(4*H*,5*H*)-diyl))diethanones **7-12 **are confirmed by FT-IR, MS, ^1^H NMR, and ^13^C NMR spectral studies and elemental analysis [21].

**Table 1:** Physical and analytical data of compounds 7-12

Compounds	X	Time Δ (h)/sonication (min)	Yield (%)Δ/sonication	m.p. (°C)	Elemental analysis (%)			*m/z *(M)^+.^molecular formula
						
					C Found (calculated)	H Found (calculated)	N Found (calculated)	
**7**	H	7/15	65/95	261	74.55 (74.65)	5.69 (5.82)	12.31 (12.44)	450 C_28_H_26_N_4_O_2_
**8**	F	7/15	70/94	233	69.02 (69.12)	4.77 (4.97)	11.41 (11.52)	486 C_28_H_24_F_2_N_4_O_2_
**9**	Cl	8/20	55/88	260	64.52 (64.74)	4.52 (4.66)	10.66 (10.79)	518, 520 C_28_H_24_Cl_2_N_4_O_2_
**10**	Br	7/15	60/95	262	55.13 (55.28)	3.82 (3.98)	9.11 (9.21)	606, 608 C_28_H_24_Br_2_N_4_O_2_
**11**	CH_3_	5/10	65/98	258	75.13 (75.29)	6.22 (6.32)	11.60 (11.71)	478 C_30_H_30_N_4_O_2_
**12**	OCH_3_	5/10	65/95	202	70.43 (70.57)	5.86 (5.92)	10.85 (10.97)	510 C_30_H_30_N_4_O_4_

### 3.2. Antimicrobial activity of 1,1'-(5,5'-(1,4-phenylene)bis(3-aryl-1H-pyrazole-5,1-(4H, 5H)-diyl))diethanones by disc diffusion method 7-12

An array of biolabile 1,1'-(5,5'-(1,4-phenylene)*bis*(3-aryl-1*H*-pyrazole-5,1-(4*H*,5*H*)-diyl))diethanones **7-12 **is tested for its antimicrobial activity by disc diffusion method against tested bacterial and fungal strains and the results are presented in Table 2. The use of 1,1'-(5,5'-(1,4-phenylene)*bis*(3-phenyl-1*H*-pyrazole-5,1-(4*H*,5*H*)-diyl))diethanone **7 **shows good zone of inhibitions against *M. luteus*. Excellent zone of inhibitions is noted against *S. aureus, β-H. streptococcus, M. luteus, B. subtilis, V. cholerae, E. coli, K. pneumonia, A. niger*, and *M. gypseum *by compound **8 **which has electron withdrawing fluoro substituent at the *para *position of the phenyl ring. The usage of compound **9 **which have electron withdrawing chloro substituent at the *para *position of the phenyl ring exhibits good zone of inhibitions against all the tested microorganisms except *S. typhii, E. coli*, and *M. gypseum*. Compound **10**, which have electron withdrawing bromo substituent at the *para *position of the phenyl ring exhibits fine zone of inhibitions against all the tested bacterial strains except *K. pneumonia*. Excellent zone of inhibition is noticed by compound **10 **against *M. gypseum*. The use of compound **11 **which have electron-donating methyl substituent at the *para *position of the phenyl ring exhibits superior zone of inhibitions against *S. flexneri, K. pneumonia, R. arrhizus*, and *M. gypseum*. Also, the use of compound **12 **which have electron donating methoxy substituent at the *para *position of the phenyl ring exerts higher zone of inhibitions against *K. pneumonia, A. niger*, and *M. gypseum*.

**Table 2:** *In vitro *antibacterial and antifungal activities of compounds 7-12 by disc diffusion method

Microorganisms	Compound 7 (ppm)			Compound 8 (ppm)			Compound 9 (ppm)			Compound 10 (ppm)			Compound 11 (ppm)			Compound 12 (ppm)		
	
	100	200	500	100	200	500	100	200	500	100	200	500	100	200	500	100	200	500
*Staphylococcus aureus*	++	+++	+++	-	++	+++	++	++++	++++	++	+++	+++	+	++	++	-	++	+++
*β-Heamolytic streptococcus*	++	++	+++	++	+++	++++	++	+++	+++	++	+++	+++	++	+++	+++	++	++	++
*Micrococcus luteus*	++	+++	++++	++	+++	++++	++	+++	+++	++	++	+++	-	++	++	++	++	++
*Bacillus subtilis*	++	+++	+++	++	++++	++++	++	+++	+++	-	++	+++	-	++	+++	-	++	++
*Salmonella typhii *	++	++	++	++	+++	+++	++	+++	++++	++	+++	++++	-	++	+++	-	++	+++
*Shigella flexneri*	-	++	++	++	++	++	++	++++	++++	++	+++	++++	++	++	++	-	++	+++
*Vibreo cholerae*	-	++	++	-	+++	+++	++	++++	++++	++	+++	++++	-	++	+++	++	++	+++
*Escherichia coli*	++	++	+++	++	+++	+++	-	+++	+++	++	++	+++	++	++	+++	++	++	++
*Pseudomonas aeruginosa*	++	++	++	++	+++	+++	++	+++	++++	++	++	+++	++	++	+++	+	++	++
*Klebsiella pneumonia*	++	++	+++	+++	+++	++++	++	+++	++++	++	++	++	++	+++	++++	++	++++	++++
*Aspergillus flavus*	-	++	+++	++	+++	+++	++	+++	++++	++	++	++	++	++	+++	-	++	++
*Aspergillus niger*	+	++	++	++	+++	+++	++	+++	+++	+	+	++	++	++	+++	++	+++	++++
*Mucor indicus*	-	++	++	+	++	++	++	+++	++++	++	++	+++	++	++	+++	+	++	++
*Rhizopus arrhizus*	-	++	++	+	++	++	++	++	+++	++	++	++	++	++	+++	++	++	+++
*Microsporum gypseum*	++	++	+++	++	+++	++++	++	++	++	++	+++	++++	++	+++	++++	++	+++	++++

(-) = inactive, (+) = weakly active(12-16 mm), (+)(+) = moderately active(17-21 mm), (+)(+)(+) = strong active(22-29 mm), (+)(+)(+)(+) = highly active(30-33 mm). At 500 ppm concentration, standard antibacterial drug, ciprofloxacin exhibits 30 ± 0.5 mm zone of inhibition against all the test bacteria and standard antifungal drug, fluconazole exhibits 20 ± 0.5 mm zone of inhibition against all the test fungi.

### 3.3. Antimicrobial activity of 1,1'-(5,5'-(1,4-phenylene)bis(3-aryl-1H-pyrazole-5,1-(4H, 5H)-diyl))diethanones by twofold serial dilution method 7-12

*In vitro *antimicrobial results by the twofold serial dilution method (Table 3) of 1,1'-(5,5'-(1,4-phenylene)*bis*(3-aryl-1*H*-pyrazole-5,1-(4*H*,5*H*)-diyl))diethanones **7-12 **show that compound **7 **exhibits good activities against *M. luteus *at a MIC value of 6.25 μg/mL. Admirable activities against *β-H. streptococcus, M. luteus, K. pneumonia*, and *M. gypseum *are displayed by compound **8 **at a MIC value of 6.25 μg/mL, whereas it displays modest activities against *S. aureus *and *B. subtilis *at a MIC value of 12.5 μg/mL. The use of compound **9 **displays higher activities against *S. aureus, S. flexneri, V. cholerae, P. aeruginosa, A. flavus*, and *M. indicus *at a MIC value of 6.25 μg/mL. Excellent antimicrobial activities are exhibited by compound **10 **against *S. typhii, S. flexneri*, and *M. gypseum *at a MIC value of 6.25 μg/mL, whereas it exhibits superior activities against *V. cholerae, E. coli*, and *P. aeruginosa *at a MIC value of 12.5 μg/mL. The use of compound **11**, which has electron donating methyl group at the *para *position of the phenyl ring, exhibits greater activities against *K. pneumonia *and *M. gypseum *at a MIC value of 6.25 μg/mL. Modest activities are displayed by compound **12 **against *A. niger *at a MIC value of 12.5 μg/mL, whereas it exhibits strong activities against *K. pneumonia *and *M. gypseum *at a MIC value of 6.25 μg/mL.

**Table 3:** *In vitro *antibacterial and antifungal activities of compounds 7-12 by twofold serial dilution method

Microorganisms	Minimum inhibitory concentration (MIC) (μg/mL)							
	
	7	8	9	10	11	12	Ciprofloxacin	Fluconazole
*Staphylococcus aureus*	50	12.5	6.25	25	200	100	25	-
*β-Heamolytic streptococcus*	50	6.25	25	25	100	200	25	-
*Micrococcus luteus*	6.25	6.25	25	25	200	200	12.5	-
*Bacillus subtilis*	50	12.5	25	25	200	200	12.5	-
*Salmonella typhii*	100	50	50	6.25	100	200	25	-
*Shigella flexneri*	200	100	6.25	6.25	25	100	12.5	-
*Vibreo cholerae*	100	25	6.25	12.5	50	100	25	-
*Escherichia coli*	50	25	50	12.5	50	100	25	-
*Pseudomonas aeruginosa*	25	100	6.25	12.5	100	50	25	-
*Klebsiella pneumonia*	25	6.25	25	200	6.25	6.25	12.5	-
*Aspergillus flavus*	50	50	6.25	200	50	100	-	12.5
*Aspergillus niger*	200	25	25	100	50	12.5	-	12.5
*Mucor indicus*	100	100	6.25	100	100	200	-	25
*Rhizopus arrhizus*	100	100	25	100	25	100	-	25
*Microsporum gypseum*	50	6.25	200	6.25	6.25	6.25	-	12.5

### 3.4. Antitubercular activity of 1,1'-(5,5'-(1,4-phenylene)bis(3-aryl-1H-pyrazole-5, 1-(4H, 5H)-diyl))diethanones by luciferase reporter phage assay method 7-12

*In vitro *antitubercular activity screening was evaluated against *M. tuberculosis *H_37_Rv, INH-resistant *M. tuberculosis *by luciferase reporter phage assay method at two different concentrations (1.00 and 2.00 mg/mL). The observed percentage inhibitions are summarized in Table 4. A compound is considered to possess antimycobacterial activity if 50% reduction in the relative light units (RLU) is observed when compared to the control using a luminometer. *In vitro *antitubercular activity results of **7-12 **show that all the synthesized compounds exhibited good activity against the tested two *M. tuberculosis *bacterial strains, namely, *M. tuberculosis *H_37_Rv and INH-resistant *M. tuberculosis*. It is observed from Table 4 that the activity of compounds get increased as the concentration of compound increases from 1.00 to 2.00 μg/mL. The percentage of reduction in RLU for the synthesized compounds is in the range of 74-88% against the tested bacterial strain *M. tuberculosis *H_37_Rv and 73-85% against the tested bacterial strain INH-resistant *M. tuberculosis*. Among the synthesized compounds, compound **8 **exhibited excellent antitubercular activity against *M. tuberculosis *H_37_Rv and the percentage of reduction in RLU for **8 **is 89%. Similarly, compound **11 **exhibited excellent antitubercular activity against INH-resistant *M. tuberculosis *and the percentage of reduction in RLU for fluorine-substituted compound **8 **is 85%. Also, fluorine substitution is commonly used in contemporary medicinal chemistry to improve metabolic stability, bioavailability, and protein-ligand interactions [28-30].

**Table 4:** *In vitro *antitubercular activity of compounds 7-12 by luciferase reporter phage assay method

Compounds	*M. Tuberculosis* H_37_Rv		INH resistant *M. tuberculosis*	
	
	1.00 (μg/mL)	2.00 (μg/mL)	1.00 (μg/mL)	2.00 (μg/mL)
**7**	74	78	73	77
**8**	85	89	79	82
**9**	82	85	79	82
**10**	79	81	78	80
**11**	81	83	82	85
**12**	82	84	80	84
**Isoniazid**	99	99	90	96

## 4. Conclusion

A clean, efficient, convenient, and economical synthesis of 1,1'-(5,5'-(1,4-phenylene)*bis*(3-aryl-1*H*-pyrazole-5,1-(4*H*,5*H*)-diyl))diethanones using ultrasound irradiation is described. The microbiological screening studies carried out to evaluate the antibacterial and antifungal potencies of the synthesized 1,1'-(5,5'-(1,4-phenylene)*bis*(3-aryl-1*H*-pyrazole-5,1-(4*H*,5*H*)-diyl))diethanones **7-12 **were clearly known from Tables 2 and 3. *In vitro *antibacterial and antifungal activities profile of substituted aromatics (X = F, Cl, Br) are more active than non-substituted aromatic ring system (X = H) of novel target compounds exerted strong antibacterial and antifungal activity against all the tested bacterial strains. Among all the tested compounds, electron withdrawing-substituted compounds **8**, **9**, and **10 **exerted moderate antimicrobial activity and the range of MIC values of **8**-**10 **are 200-6.25 μg/mL. Among the synthesized compounds, compound **8 **against *M. tuberculosis *and compound **11 **against INH-resistant *M. tuberculosis *exhibited the percentage of reduction in RLU at 89 and 85%, respectively. Further development of this group of 1,1'-(5,5'-(1,4-phenylene)*bis*(3-aryl-1*H*-pyrazole-5,1-(4*H*,5*H*)-diyl))diethanones may lead to compounds with better pharmacological profile than standard antibacterial, antifungal, and antitubercular drugs that are under progress.

## Competing interests

The authors declare that they have no competing interests.
